# Supplementary material for: Decreased dihydroartemisinin-piperaquine protection against recurrent malaria associated with Plasmodium falciparum plasmepsin 3 copy number variation in Africa
Source: Nat Commun. 2025 Mar 18;16:2680. doi: 10.1038/s41467-025-57726-5 (PMC11920258; doi:10.1038/s41467-025-57726-5)
Supplement: Supplementary file 1 — Supplementary Information [file 41467_2025_57726_MOESM1_ESM.pdf]

Supplementary Material

Supplement to: Decreased dihydroartemisinin-piperaquine protection against recurrent malaria associated with *plasmepsin 2/3* copy number variation in Africa

The authors' full names and academic degrees are as follows:

Leyre Pernaute Lau, PhD; Mario Recker, PhD; Mamadou Tékété, PhD; Tais Nóbrega de Sousa, PhD; Aliou Traore, MSc; Bakary Fofana, MD; Kassim Sanogo, MSc; Ulrika Morris, PhD; Juliana Inoue, PhD; Pedro Eduardo Ferreira, PhD; Nouhoum Diallo, MD; Jürgan Burhenne, PhD; Issaka Sagara, PhD; Alassane Dicko, PhD; Maria Isabel Veiga, PhD; Walter Haefeli, MD; Anders Björkman, MD, PhD; Abdoulaye A Djimde, PhD, Steffen Borrmann, MD, PhD; Jose Pedro Gil, PhD.

Index

SUPPLEMENTARY TABLE 1. CHARACTERISTICS OF THE PATIENTS AT RECRUITMENT. 3

SUPPLEMENTARY TABLE 2. DOSING OF PATIENTS. 3

SUPPLEMENTARY TABLE 3. MOLECULAR METHODOLOGY AND PRIMER SEQUENCES 3

SUPPLEMENTARY TABLE 4. WORKFLOW OF THE ANALYSIS 5

SUPPLEMENTARY TABLE 5. REASONS FOR WITHDRAWAL OF PATIENTS. 5

SUPPLEMENTARY FIGURE 1. PFPM2 COPY NUMBER VARIATION DISTRIBUTION 6

SUPPLEMENTARY FIGURE 2. PFPM3 COPY NUMBER DISTRIBUTION IN ARTESUNATE-AMODIAQUINE (ASAQ) TREATMENT ARM 6

SUPPLEMENTARY FIGURE 3. INTER-INFECTION PERIOD DISTRIBUTION OF PFCRT MUTANT AND WILDTYPE INFECTIONS 7

SUPPLEMENTARY TABLE 1. CHARACTERISTICS OF THE PATIENTS AT RECRUITMENT.

**Characteristic**	**Dihydroartemisinin-piperaquine (N = 225)**	**Artesunate-amodiaquine (N = 224)**
**Median age, range – yr**	8.2 (0.8 - 16.0)	8.0 (0.7 - 17.4)
**Female sex – no. (%)**	113 (50.2)	105 (46.9)
**Median asexual *Plasmodium falciparum* parasitemia – no./µL**	17,000 (0 - 154,000)	14,600 (0 - 199,000)
**Median body weight, range – kg**	20.3 (7.3 - 63.2)	20.8 (6.0 - 51.1)
**Mean hemoglobin concentration – g/dL**	10.3 (7 – 13.2)	10.2 (7.1 – 13.4)

SUPPLEMENTARY TABLE 2. DOSING OF PATIENTS.

**Study drug**	**Formulation**	**Number of tablets**	**Body weight**
Dihydroartemisinin-piperaquine	20:160 mg tablet	0.5	5 to <7
	20:160 mg tablet	1	7 to <13
	40:320 mg tablet	1	13 to <24
	40:320 mg tablet	2	24 to <36
	40:320 mg tablet	3	36 to <75
	40:320 mg tablet	4	≥75
Artesunate-amodiaquine	25:67.5 mg tablet	1	5 to <9
	50:135 mg tablet	1	9 to <18
	100:270 mg tablet	1	18 to <36
	100:270 mg tablet	2	≥36

SUPPLEMENTARY TABLE 3. MOLECULAR METHODOLOGY AND PRIMER SEQUENCES

Copy Number Variation Analysis

**Gene**	**Primers**	**Cycling requirements**
pfpm II	Forward 5'-TGGTGATGCAGAAGTTGGAG-3'<br>Reverse 5'-TGGGACCCATAAATTAGCAGA-3'	95°C 15min; 45cycles 95°C 15sec; 63°C 20sec; 72°C 20'sec
pfpm III	Forward 5'-CCACTTGTGGTAACACGAAATTA-3′<br>Reverse 5'-TGGTTCAAGGTATTGTTTAGGTTC-3′	95°C 10min; 50cycles 95°C 15sec; 58°C 1min
βtubulin	Forward 5'-TGATGTGCGCAAGTGATCC-3′<br>Reverse 5'-TCCTTTGTGGACATTCTTCCTC-3′	95°C 15min; 45cycles 95°C 15sec; 63°C 20sec; 72°C 20'sec

**Probe Name**	**5' DYE**	**Sequence 5'-3'**	**3' Quencher**
btub	HEX	TAGCACATGCCGTTAAATATCTTCCATGTCT	BHQ-1
Pfpm3	FAM	CCAACACTCGAATATCGTTCACCAA	BHQ-1

Pfpm2 copy number was determinate by SYBR-green based quantitative PCR (ThermoFisher Scientific, Waltham®, MA, USA) in a protocol modified from the one previously described by Witkowski et al[8]. We had optimized the TaqMan-based protocol of Ansbro et al. (2020) (*13*) to our BioRad qPCR system for the analysis of copy number variation in *pfpm3*. For each gene, 4µL of extracted DNA was used as template in a final volume of 21 µL with 1XSsoadvanced Universal Probes Supermix (Bio-Rad, Hercules, CA), 300nM of the forward and reverse primers and 100nM of the probes.

All procedures were conducted in triplicate using pf *β-tubulin* gene as the internal single copy control gene. 3D7 clone was used as a parallel 1 copy control calibrator, and a previously designed genetically modified clone with 2 copies of *pfpm2* gene used as control for multicopies. For both genes, CNV was calculated as previously described (*7*) following the formula: CNV=2-ΔCt. Amplifications were carried out in triplicate. Samples with Ct-values >35 or a standard deviation greater than 0.5 in the triplicate Ct values were excluded. A CNV>1.5 was considered pure multicopy.

Pfcrt Single Nucleotide Polymorphisms analysis

***Pfcrt* SNPs**	**Primer**	**MasterMix**
**C101F**	Forward 5'-GTTCTTGTCTTGGTAAATGTGC-3'	All PCR reactions contained 1 × Taq polymerase reaction buffer, 2.5–3 mM magnesium chloride, 2 mM dNTP, 0.5–1 µM of each primer and 1.25 units of Taq DNA polymerase (Promega Corporation, USA)
	Reverse 5'-GAATTTCCCTTTTTATTTCCAAATAAGGAATA-3'	
**F145I**	Forward 5'-GAACGACACCGAAGCTTTAATTT-3'	
	Reverse 5'-AAGCAGAAGAACATATTAATAGGAATAC-3'	
**N326S**	Forward 5'-AGAAAACCTTCGCATTGTTTTCCTTC-3'	
	Reverse 5'-TCTTGTATGTATCAACGTTTTTCATCC-3'	
	NestForward 5'-cgccctccgcccgacggacgAGAAAACCTTCGCATTGTTTTCCTTC-3'	
	NestReverse 5'-ccgccctccgcccgacggacgTGTGATAACTTGATAACCAGCTATGTAAG-3'	
**I356T**	Forward 5'-TCGACAAATTTTCTACCATGACATAT-3'	
	Reverse 5'-GGCTAAGAATTTAAAGTAATAAGC-3'	
	NestForward 5'-tcgtcggcagcgtcagatgtgtataagagacagGTTAGTTGTATACAAGGTCCAGCAC-3'	
	NestReverse 5'-tcgtcggcagcgtcagatgtgtataagagacagGGCTAAGAATTTAAAGTAATAAGC-3'	
**K76T**	Forward 5'-GCGCGCGCATGGCTCACGTTTAGGTGGAG-3'	
	Reverse 5'-GGGCCCGGCGGATGTTACAAAACTATAGTTACC-3'	
	NestForward 5'-TGTGCTCATGTGTTTAAACTT-3'	
	NestReverse 5'-CAAAACTATAGTTACCAATTTTG-3'	

Restriction enzymes for PCR-RFLP: ApoI (K76T), MseI (N326S), BfaI (I356T). *Pfcrt* C101F and F145I SNPs were analyzed by Sanger Sequencing.

SUPPLEMENTARY TABLE 4. WORKFLOW OF THE ANALYSIS

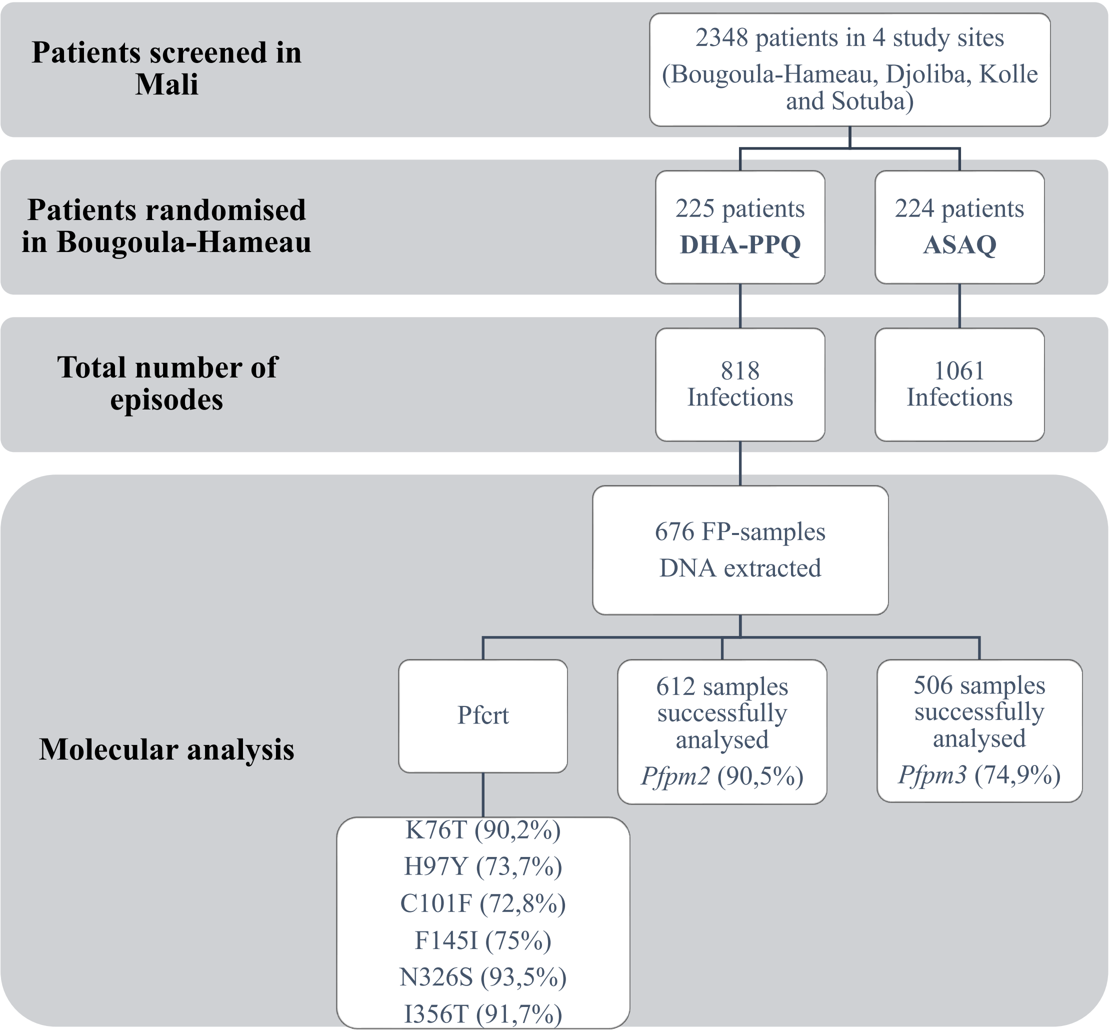

FP: Filter paper

Chart flow that describes the complete analysis of the samples from the original trial performed in Mali.

SUPPLEMENTARY TABLE 5. REASONS FOR WITHDRAWAL OF PATIENTS.

Reason	Dihydroartemisinin-piperaquine (N = 225)	Artesunate-amodiaquine (N = 224)
**No longer willing to participate**	6	8
**Lost to follow-up**	2	2

**Treatment failure**	1	-
**Death**	1	-
**Other reasons**	4	1

SUPPLEMENTARY FIGURE 1. *PFPM2* COPY NUMBER VARIATION DISTRIBUTION

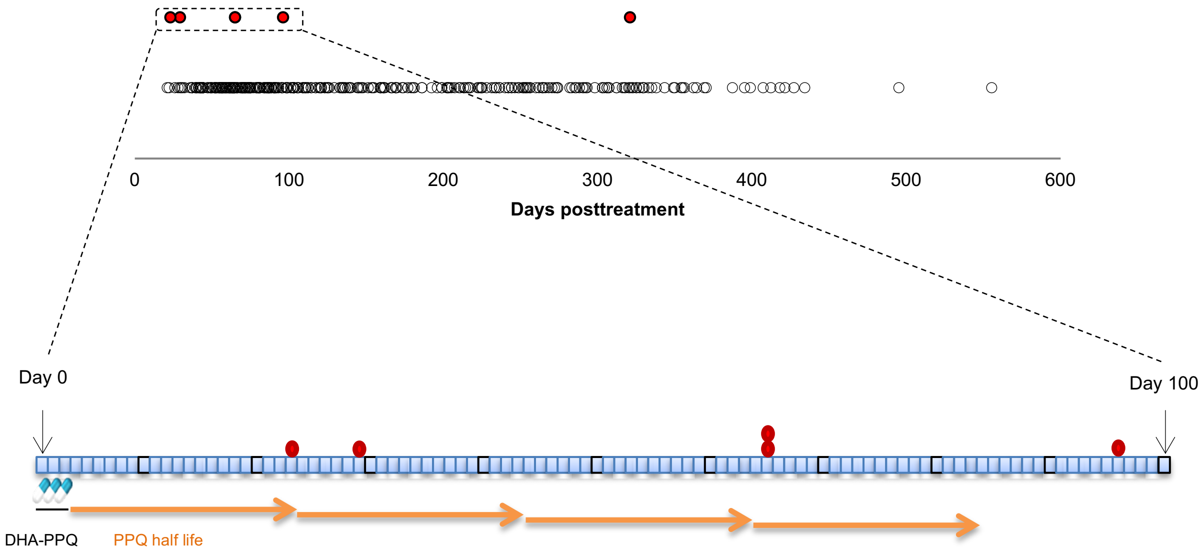

Timeline distribution of *Plasmodium falciparum pfpm2* copy number status during DHA-PPQ treatment follow-up. Circles represent infections; white circles indicate *pfpm2* single copy infections and red circles indicate *pfpm2* multicopy infections.

SUPPLEMENTARY FIGURE 2. *PFPM3* COPY NUMBER DISTRIBUTION IN ARTESUNATE-AMODIAQUINE (ASAQ) TREATMENT ARM

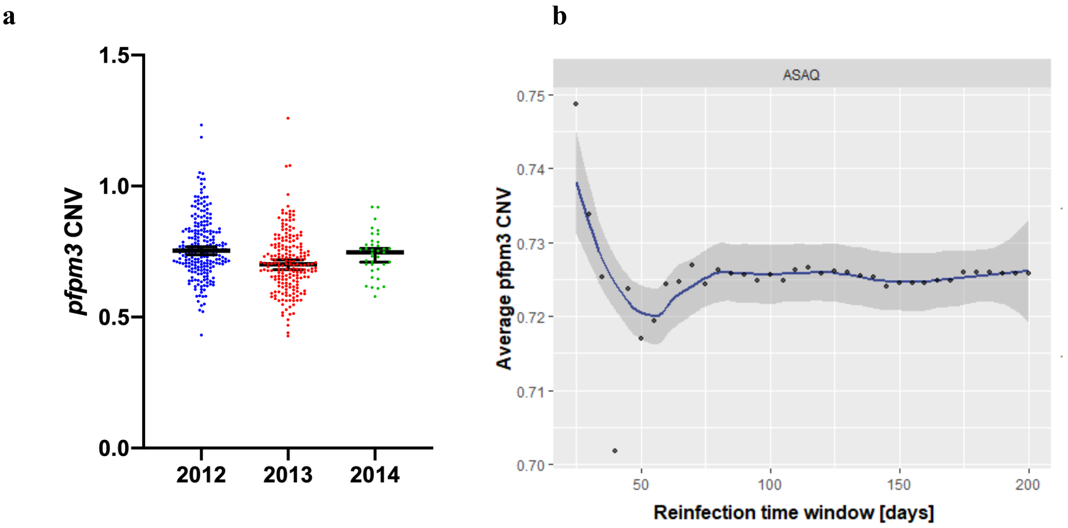

Dots represent infections, solid lines represent median values of *pfpm3* CNV with 95% CI (2012: 0.75 [0.74,0.77], 2013: 0.71 [0.68, 0.72]; 2014: 0.74 [0.71, 0.76]). A total of 473 infections were analyzed from January 2012 until December 2014 in Bougoula-Hameau study site (a). *Pfpm3* gene dose effect *versus* time to reinfection after ASAQ treatment (b).

SUPPLEMENTARY FIGURE 3. INTER-INFECTION PERIOD DISTRIBUTION OF PFCRT MUTANT AND WILDTYPE INFECTIONS

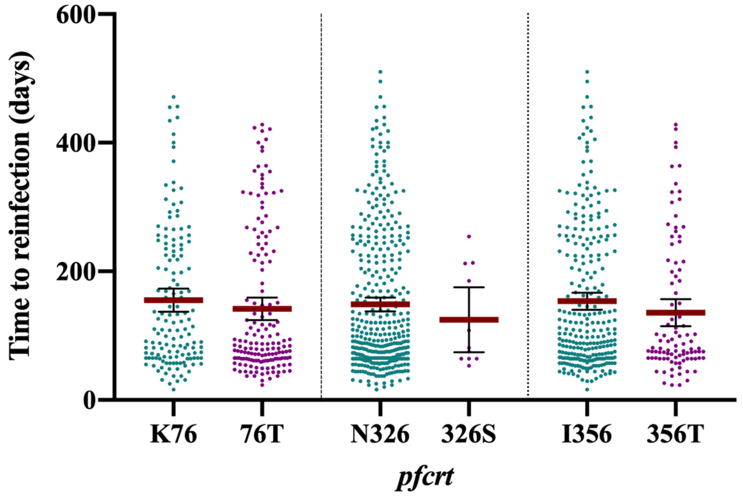

Dots represent infections, solid red lines represent mean values of interinfection period with 95% CI. Mutant SNPs were associated with decreased times to re-infection compared with the wildtype infections (interinfection period (days): 155.4 *vs* 141.8; 148.5 *vs* 124.8; 153.5 *vs* 135.8, for *pfcrt* 76, 326 and 356 SNPs, respectively). Out of the remaining *pfcrt* SNPs analyzed (H97Y, C101F and F145I) only wildtype infections were identified.
